# GECluster: a novel protein complex prediction method

**DOI:** 10.1080/13102818.2014.946700

**Published:** 2014-10-17

**Authors:** Lingtao Su, Guixia Liu, Han Wang, Yuan Tian, Zhihui Zhou, Liang Han, Lun Yan

**Affiliations:** ^a^ College of Computer Science and Technology, Jilin University, Changchun, P. R. China; ^b^ Key Laboratory of Symbolic Computation and Knowledge Engineering of Ministry of Education, Jilin University, Changchun, P. R. China; ^c^ College of Computer Science and Information Technology, Northeast Normal University, Changchun, P. R. China

**Keywords:** protein complex, evolution, gene expression value, PPI, GO, core and attachment protein

## Abstract

Identification of protein complexes is of great importance in the understanding of cellular organization and functions. Traditional computational protein complex prediction methods mainly rely on the topology of protein–protein interaction (PPI) networks but seldom take biological information of proteins (such as Gene Ontology (GO)) into consideration. Meanwhile, the environment relevant analysis of protein complex evolution has been poorly studied, partly due to the lack of high-precision protein complex datasets. In this paper, a combined PPI network is introduced to predict protein complexes which integrate both GO and expression value of relevant protein-coding genes. A novel protein complex prediction method GECluster (Gene Expression Cluster) was proposed based on a seed node expansion strategy, in which a combined PPI network was utilized. GECluster was applied to a training combined PPI network and it predicted more credible complexes than peer methods. The results indicate that using a combined PPI network can efficiently improve protein complex prediction accuracy. In order to study protein complex evolution within cells due to changes in the living environment surrounding cells, GECluster was applied to seven combined PPI networks constructed using the data of a test set including yeast response to stress throughout a wine fermentation process. Our results showed that with the rise of alcohol concentration, protein complexes within yeast cells gradually evolve from one state to another. Besides this, the number of core and attachment proteins within a protein complex both changed significantly.

## Introduction

Protein complexes are groups of proteins that interact with each other; consequently, they usually form dense clusters in protein–protein interaction (PPI) networks. Protein complexes are important molecular entities in cells. Predicting protein complexes can help in the understanding of cellular components and function as well as in research on the evolution between protein complexes. Researching protein complex evolution due to changes in the living environment surrounding cells is of great importance in unveiling the secrets of cell organization and function.[1] Recently, a large amount of protein interactions has been produced, which makes it possible to predict protein complexes from PPI networks.

The prediction of protein complexes from PPI networks has long been studied and the methods mainly fall within the following three categories: (1) methods based on seed node expansion,[2,3] which predict protein complex in two stages: first, they identify seed nodes and second, they expand the source nodes. A different strategy used in seed node selection and expansion affects those methods significantly. (2) Methods based on hierarchical clustering,[4,5] which represent the entire network in a tree-like structure and find protein complexes by cutting the whole tree at different levels. (3) Heuristic methods,[6] which usually have much higher accuracy due to introducing additional information about proteins besides the topology of PPI networks. However, all these methods mainly rely on the topology of PPI networks. Seldom are the roles that proteins play in the cells considered. Recently, some researchers have begun to acknowledge that by introducing biological information of proteins into protein complex prediction, the accuracy of several methods can be improved. As a result, methods like those described in [7,8] can achieve much higher accuracy. However, all the methods above ignore a basic concept that PPI networks do not remain constant but experience dynamic changes according to the changes in the cellular living environment.

At a specific time point, only certain proteins that are needed are translated. Therefore, the protein complex prediction should be based on essentially translated proteins and nonessential ones should better be excluded. Besides, more biological information such as Gene Ontology (GO) and gene expression values of corresponding proteins can be taken into consideration when selecting seed node proteins.

In this paper, a combined PPI network is introduced to predict protein complexes, which integrates both GO and the expression value of relevant protein-coding genes. The combined PPI network which is constructed by the dynamic PPI network is a weighted PPI network and the weight corresponds to the number of shared GO terms between proteins. The dynamic PPI network is constructed by the PPI network while removing untranslated proteins. We propose a novel protein complex prediction method, GECluster, based on a seed node expansion strategy, in which a combined PPI network is utilized. GECluster was applied to a combined PPI network and it predicted more credible complexes than peer methods. In order to study the protein complex evolution within cells due to changes in the living environment surrounding cells, we applied GECluster to seven combined PPI networks constructed using gene expression data of yeast response to stress throughout a wine fermentation process. According to our study, with the rise of alcohol concentration, protein complexes within yeast cells gradually evolve from one state to another and the number of core and attachment proteins within protein complexes both changed significantly.

## Materials and methods

### Static PPI network and dynamic PPI network

**Figure 1.**
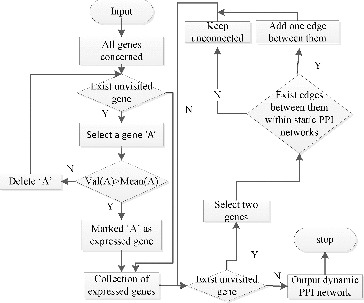
Flowchart diagram on how to construct dynamic PPI networks. Note: *val*(*A*) represents the expression value of gene ‘A’; *Mean*(*A*) represents the mean expression value of gene ‘A’, which is calculated by computing the average expression value of gene ‘A’ at different time points.

Static PPI networks, also called PPI networks, have been widely used in protein complex prediction researches.[9,10] A static PPI network contains all the PPIs in an organism, regardless of when and where the interactions occur. In other words, a static PPI network is the union set of PPIs. Dynamic PPI networks are a subset of the static PPI networks and are constructed by removing redundant PPIs from a static PPI network, leaving only interactions needed. This is reasonable because at a certain time point only certain proteins that are needed are expressed in a cell. In this paper, we constructed a dynamic PPI network by refining static PPI networks using time series gene expression data. The steps are shown in Figure 1. (For an example, see Section 1.1 in the Online Supplementary Appendix.)

### GO and GO slims

The GO [11] project is an extensive bioinformatics initiative aiming to standardize the representation of gene and gene product attributes across species and databases. As demonstrated in [8,12], a general feature of the proteins in a protein complex is that they commonly share one or more GO term annotations. GO slims are a trimmed version of GO and give a much broader overview of ontology content than GO, which makes them particularly useful for giving a summary of the results of GO annotation of a genome when broad classification of proteins is needed.[1] The annotation of GO slims mainly includes three aspects: biological processes (BP), molecular functions (MF) and cellular components (CC), each of which contains a fixed number of GO terms. In this paper, we use GO slims to annotate each protein in the dynamic PPI network and, as a result, for each GO term we obtain a protein set denoted as GO-Protein-Set. Proteins within the same GO-Protein-Set share similar GO annotations.

### Combined PPI network construction

**Figure 2.**
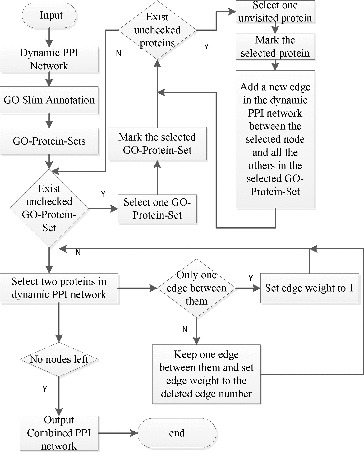
Flowchart diagram on how to construct combined PPI networks.

A combined PPI network is a weighted dynamic PPI network and the weight on the edge represents the common GO terms shared by the two endpoint proteins. We construct the combined PPI network as shown in Figure 2. (For an example, see Section 1.2 in the Online Supplementary Appendix.)

### Function similarity

Function similarity measure between two arbitrary proteins, also called *FS_Weight*,[13] has been widely used in protein complex prediction. In this paper, we also use *FS_Weight* to measure the function similarity between two proteins. The *FS_Weight* between proteins *i* and *j* is defined as follows:(1) 
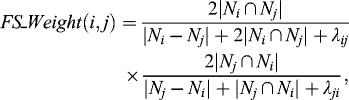
where *N_i_* denotes the degree of protein *i*; |*N_i_∩N_j_*| denotes the common neighbours between proteins *i* and *j*, including *i* and *j*; |*N_i_ − N_j_*| denotes the number of proteins who are neighbours of protein *i* but not of *j*; and λ*_ij_* is defined as follows:(2) 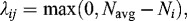
where *N*_avg_ denotes the average degree of all the proteins in the dynamic PPI network; and λ*ji* can be defined correspondingly.

In this paper, if *FS_Weight* ≥ *FS_Weight*_min_, where *FS_Weight*_min_ is a threshold value, we consider the proteins to have strong similarity. If two proteins have strong similarity, this does not necessarily mean that they would fall into one and the same cluster. In order to determine whether they should be clustered together, a *Cluster_ Coefficient* index was used. *Cluster_ Coefficient* is defined as(3) 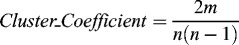
where *m* is the total edges in the cluster; and *n* (*n* ≥ *2*) is the total nodes in the cluster.

When adding a protein into a cluster, two criteria should be satisfied. One is that it should have strong functional similarity with all the proteins already in the cluster; the other one is that the *Cluster_Coefficient* should not decrease as a result of the inclusion of that protein.

### GECluster algorithm

**Figure 3.**
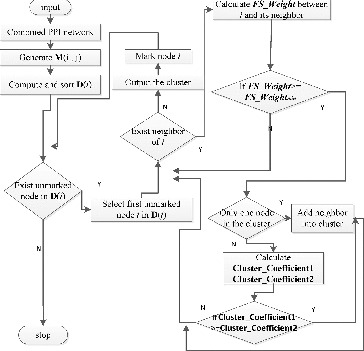
Flowchart diagram of the GECluster algorithm. Notes: *M*(*i*,*j*) is the weighted matrix of combined PPI network whose elements represent the edge weight value in combined PPI network. *D*(*i*) is an array, with element values ordered from large to small and the value represents the degree of network node. *Cluster_Coefficient1* represents the *Cluster_Coefficient* value of the new cluster before the selected node is included. *Cluster_Coefficient2* represents the *Cluster_Coefficient* value of the new cluster after the selected node is added into the cluster.

The GECluster algorithm uses combined PPI networks and works as shown in Figure 3.

### Datasets

Two time course gene expression datasets were used in this paper: one is a training set and the other one is a test set. The time series gene expression data in the training set were downloaded from Gene Expression Omnibus (GEO).[14] The accession number is GSE4259, which is a study of fermentation at different concentrations of sucrose. The time course gene expression data of *Saccharomyces cerevisiae* responding to stress throughout a 15-day wine fermentation (downloaded from GEO,[15] accession number is GSE8536) was used as test set. The main study of GSE8536 is based on expression measurements at 0.5%, 2%, 3.5%, 7% and 10% ethanol (corresponding to roughly 24, 48, 60, 120 and 340 h). Experiments were done in triplicate (biological replicates). This accession also includes the files at 1 and at 12 h for completeness (also in triplicate). The DIP (Database of Interaction Proteins) database is composed of three linked tables: a table of protein information, a table of PPIs and a table describing details of experiments detection the PPIs. The experimental article table can be used to evaluate the quality of an interaction base on the particular experiments performed.

The static PPI dataset was downloaded from DIP [16] (updated on 07/07/2013). The data have been curated both manually by expert curators and also automatically, using computational approaches that utilize the knowledge about the PPI networks extracted from the most reliable, core subset of the DIP data. After deleting self-loops and duplicated edges, the static PPI network contains 4976 nodes and 21,937 edges. In order to validate our method, a benchmark protein complex dataset was used. The reference set comprises 350 hand-curated consensus complexes. We kept only those complexes with a size no less than 3, and as a result only 182 complexes were left. Since we consider dynamic PPI networks in this study, some proteins in the benchmark datasets may not translate. Therefore when evaluating the performance of our methods, we curated the benchmark dataset according to expressed genes. As a result, only 76 complexes were left.

### Evaluation methods

We calculated *Recall* (sensitivity) and *Precision* at complex level by matching generated clusters with reference complex sets. Let *A* be a generated complex, *B* be a reference complex, *V_A_* be the set of proteins contained in *A* and *V_B_* be the set of proteins contained in *B*. The matching score between *A* and *B* is defined as follows:(4) 
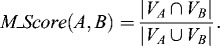



Given a threshold value *M_thres*, if *M_Score(A, B) ≥ M_thres*, then proteins *A* and *B* match each other. In most of our experiments, we set *M_thres* = 0.2, in line with many other methods.[1,17] Given a set of reference complexes *C* = (*C*_1_, *C*_2_, *C*_3_,…, *C_m_*) and a set of predicted complexes *P* = (*P*_1_, *P*_2_, *P*_3_,…, *P_n_*), *Recall* and *Precision* at complex level are defined as follows:(5) 
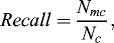

(6) 
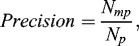
where *N_c_* and *N_p_* represent the number of complexes in the reference and the predicted protein complex data sets, respectively; *N_mc_* is the number of protein complexes in the reference complex set matched by the complexes in the predicted datasets; and *N_mp_* is the number of protein complexes in the predicted complex set matched by the complexes in the reference dataset. To obtain an overall measurement of our method, we introduced *F*1, which is defined as follows:(7) 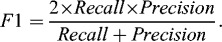



In order to further verify our results, we used the Database for Annotation, Visualization and Integrated Discovery (DAVID) [18] to annotate each protein in the predicted complex sets.

## Results and discussion

### Combined PPI network constructed based on training set

**Figure 4.**
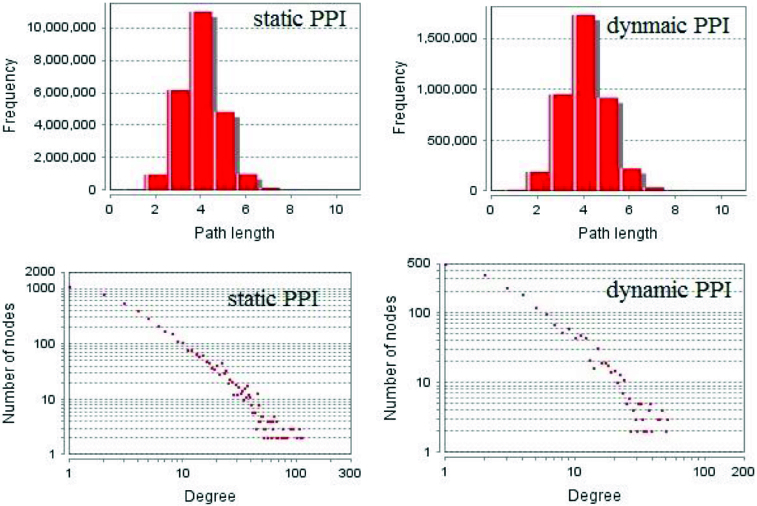
Degree and path length distribution of dynamic PPI network inferred and static PPI network.

According to the methods described above, we first constructed dynamic PPI networks by removing any untranslated proteins from the static PPI network by using gene expression datasets. We found that of the 4976 proteins in the static PPI networks, only 2078 were demonstrated to be expressed (41.8%) with 6823 edges between them. It is well known that PPI networks, as well as many other networks within biological bodies, are all complex networks which have the characteristics of being ‘scale-free’ and ‘small world’. We compared the degree and path length distribution of the dynamic PPI network we inferred with the static PPI network. The result is shown in Figure 4.

As shown in Figure 4, the dynamic PPI network we inferred also exhibits the characteristics of complex networks just as static PPI networks. We used GO slim to annotate each protein-coding gene in the dynamic PPI network. The combined PPI network was constructed by integrating the information of the dynamic PPI network and the GO annotation results. The detailed information is shown in Table 1.

**Table 1.** Network information.

Network name	Node number	Edge number
Static PPI network	4971	21937 (no self-loop)
Dynamic PPI network	2078	6823
Combined PPI network	Node number	Added edge number
	2078	1475155

### Complex prediction and comparison with other methods

In the next step, the GECluster algorithm was applied to the combined PPI network inferred above. We compared our algorithm with MCODE [3] and CFinder,[19] which were applied to the dynamic PPI network we constructed (see above). The MCODE algorithm is the earliest and most classical protein complex prediction method based on the seed node expansion strategy. CFinder has been proved to outperform other methods in finding potential protein complexes within PPI networks.[20]

**Figure 5.**
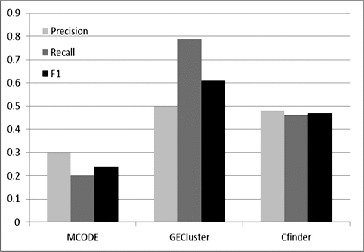
Prediction accuracy comparison between GECluster, MCODE and CFinder.

The results in Figure 5 show that by introducing the combined PPI network, our method can achieve much higher accuracy than the other peer methods. The reason obviously lies in the fact that we refined the static PPI networks by using gene expression datasets and as a result only expressed genes were selected for later use. Moreover, we used GO Slims to annotate each protein. By running this function, similar proteins are approximately grouped before our algorithm starts. Compared with our method, the other two methods all act on static PPI networks which contain genes that are actually not expressed, and thus they achieved very low prediction accuracy.

As a further verification of our results, we annotated each protein in the predicted complex sets by using the DAVID database.[19] Table 2 lists the annotation result, and again the best result of MCODE was selected and the parameter *FS_Weight*_min_ = 0.5 was set for GECluster. As seen from Table 2, our method can yield more functionally similar protein complexes with low *P*-values.

**Table 2.** Complex function annotates results.

Algorithm	Complex	Function term	Size	Annotation score	*p*-value
GECluster	CDC16, CDC26, APC11, CDC27, DOC1, APC2	Cyclin catabolic process	6	100%	3.5E−14
	RRP43, SKI6, CSL4, RRP45, RRP46 DIS3, RRP4	Exosome	7	100%	9.3E−18
	RPB8, RPC25, RPC34, RPC17	DNA-directed RNA polymerase III complex	4	100%	8.2E−8
	ORC3, ORC1, ORC2, ORC5, ORC6, ORC4	Origin recognition complex	6	100%	3.5E−16
	KTI12, ELP3, ELP2, IKI1	tRNA wobble uridine modification	4	100%	1.4E−7
	SWC4, YAF9, HTZ1, SWC7, SWR1	Chromatin regulator	5	100%	4.8E−8
	SAS5, SAS4, SAS2	SAS acetyltransferase complex	3	100%	2.8E−7
	MPE1, YSH1, YTH1, FIP1, PTA1, CFT1	mRNA cleavage and polyadenylation specificity factor complex	6	100%	2.6E−13
	CLF1, PRP19, PRP45, CEF1	Spliceosome	4	100%	5.2E−7
	SYF1, SYF2, ISY1, PRP19, CLF1	First spliceosomal transesterification activity	5	100%	1.6E−11
MCODE	MED2, GAL11, MED8, ROX3, MED7, SRB4, SPT15, SRB5	Srb-mediator complex	8	85.7%	1.2E−13
	CDC26, CDC16, CDC27, APC2, DOC1, APC11	Cyclin catabolic process	6	100%	3.5E−14
	GAS3, GPI8, NSG1, PHO86, GPI2, SUR2, BSD2	Endoplasmic reticulum	7	85.7%	4.5E−5
	RIX1, IPI3, BUD20, NOG2, SDA1, ARX1, NOP15	Ribosomal large subunit biogenesis	7	85.7%	3.4E−9
	TUM1, NCS6, UBA4, NCS2	Wobble position uridine thiolation	4	100%	5.2E−10
	GIM5, YKE2, TUB4, PAC10	Tubulin complex assembly	4	75%	5.3E−6
	HRR25, LTV1, RIO2, TSR1, NUG1, RPS28B, EDC3	Ribonucleoprotein complex biogenesis	7	100%	2.8E−7
	MED2, GAL11, MED8, ROX3, MED7, SRB4, SPT15, SRB5	Srb-mediator complex	8	85.7%	1.2E−6
	ELP4, RPO21, RPB5, TFG2, RPB7, RPB2, RPB9, RPB3, RPB4, IKI3, ELP3, ELP2, IKI1, ELP6	DNA-directed RNA polymerase II, core complex	14	50%	2.2E−13

Note: Element list in complex column represents the complex predicted by the corresponding method. Each complex consists of several proteins labelled by the gene names (named by Committee of Human Gene Nomenclature) and separated by commas. Function term describes the function of each complex. Annotation score is calculated by counting the number of proteins that have the annotated function term, out of all the proteins in the protein complex.

### Parameters discussion

**Figure 6.**
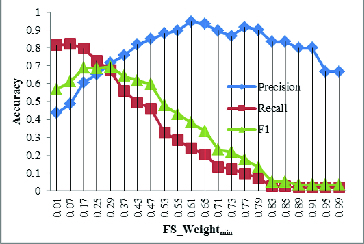
Influence of *FS_Weight*_min_ on *Precision*, *Recall* and *F*1.

Protein complex evolution has been poorly researched partly because of the lack of high-quality protein complex datasets. The GECluster algorithm, by making use of combined PPI networks, can achieve much higher prediction accuracy, which can be used for protein complex prediction. In order to determine the best parameter of GECluster, in this part of our study, we varied *FS_Weight*_min_ with steps of 0.01 starting from 0.01 to 1. The corresponding changes in *Precision*, *Recall* and *F*1 are shown in Figure 6.

The results revealed that when *FS_Weight*_min_ = 0.25, the *F*1 value is much higher (Figure 6). That is why, in the subsequent evolution analysis of protein complexes, we set *FS_Weight*_min_ = 0.25.

**Figure 7.**
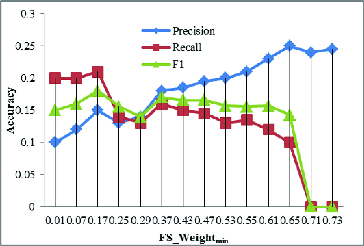
Influence of *FS_Weight*_min_ on *Precision*, *Recall* and *F*1.

In this paper, we introduced dynamic PPI networks and based on dynamic PPI networks we proposed to construct a combined PPI network which is a weighted PPI network. As we can see from the previously section, by introducing combined PPI networks, our GECluster algorithm achieved much higher accuracy in finding protein complexes within PPI networks. As a next step, we analysed the influence of *FS_Weight*_min_ on the prediction accuracy when the combined PPI network was constructed from static PPI networks rather than from dynamic PPI networks. The results are shown in Figure 7.

Compared to the accuracy values shown in Figure 6, the values in Figure 7 are much smaller. This demonstrates that, by introducing a dynamic PPI network in combined PPI network construction, the prediction accuracy can be improved.

### Protein complex evolution relationship research

**Figure 8.**
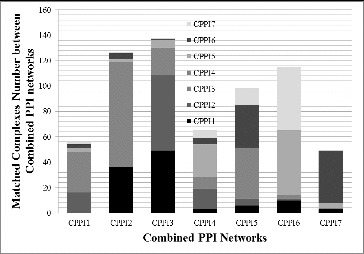
Relationships among protein complexes between different PPI networks.

By using the training set, we successfully identified the best parameter value for GECluster when applied to combined PPI networks for protein complex prediction. Then, we further analysed how protein complexes within yeast cells evolve according to the changes in the alcohol concentration in the medium during the cultivation process. As a result, seven dynamic PPI networks and their corresponding combined PPI networks were constructed by analysing data values in test datasets. The details of these networks are shown in Table 3. GECluster was applied to the seven combined PPI networks shown in Table 3, and the number of complexes predicted is also given in Table 3. The relationships among protein complexes between different PPI networks are presented in Figure 8.

**Table 3.** Information about the dynamic PPI networks and combined PPI networks inferred from test datasets and the complex numbers predicted.

Dynamic network	Node number	Edge number	Clustering coefficient	Network diameter	Characteristic path length	Combined network	Added edges	Complex numbers
DPPI1	2227	7230	0.126	12	4.226	CPPI1	1,612,056	199
DPPI2	2123	6954	0.101	10	4.067	CPPI2	1,404,088	134
DPPI3	2124	6624	0.121	12	4.177	CPPI3	1,419,210	174
DPPI4	1945	4987	0.103	12	4.443	CPPI4	1,238,366	101
DPPI5	2081	5067	0.075	11	4.445	CPPI5	1,386,405	87
DPPI6	1921	4467	0.081	11	4.564	CPPI6	1,138,527	95
DPPI7	1506	3117	0.06	12	4.709	CPPI7	698,317	70

Note: Node number is the protein number in the dynamic network; clustering coefficient is a measure of the degree to which nodes in a graph tend to cluster together; network diameter is the average minimum distance between pairs of nodes; characteristic path length is defined as the average number of edges in the shortest paths between all vertex pairs; added edges is the number of edges added when constructing combined PPI network; complex numbers are the number of complexes predicted.

The results in Figure 8 indicate that with the increase of alcohol concentration, the protein complexes within yeast cells change gradually. For example, the complexes in CPPI1 have the strongest relationship with the complexes in CPPI3 and CPPI4 but seldom have relations with complexes in other networks. The complexes in CPPI7 have most interactions with the complexes in CPPI6 but rarely interact with complexes in CPPI1, CPPI2 and so on. This illustrates how complexes evolve gradually from one state to another in order to best adapt to the changes in the living environment. The number of complexes decreased from CPPI1 to CPPI7, which was in agreement with the real-life experimental data.

At the beginning of fermentation, yeast cells multiply rapidly and, as a result, many protein complexes are synthesized. On the contrary, with the increase in alcohol concentration, yeast cells have to reduce their energy consumption and limit other non-essential activities, resulting in only a few complexes being synthesized. According to Gavin et al.,[21] the proteins within complexes can be classified into core and attachment proteins. Core proteins are those that are always present in all isoforms and execute the main functions, whereas attachment proteins are present only in some of the isoforms and act as modifiers of the complex's function.[22] Many researchers believe that core proteins evolve slower than attachment proteins, as core proteins are main functional elements,[23,24] which is in accordance with the proposal that functionally important genes should evolve slower than less important genes. However, a recent study by Chakraborty and Ghosh [22] revealed that core proteins evolve faster than attachment proteins in spite of their higher multi-functionality. The authors observed that attachment proteins play a role in a higher number of protein complexes than core proteins and, consequently, have an increased expression level, which is the main determining factor for the protein evolution rate.

In this paper, by introducing dynamic and combined PPI networks as well as the GECluster algorithm, we were able to identify the protein complexes within PPI networks precisely. Since previously performed protein complex evolution research is based on the protein complexes predicted by using low accuracy methods, it may not be completely correct. In this part of our study, the core and attachment protein dataset of [22] was used to analyse their evolutionary relationship. We count the core and attachment protein number in each network complex dataset. The protein complex number is defined as the number of protein complexes in which a protein subunit belongs. We also made a statistical assessment of the protein complex numbers of both the core and the attachment proteins. Importantly, previous studies are almost all based on static PPI networks but seldom consider complex dynamic changes. According to our study, the number of core proteins decreased significantly as compared to that of attachment proteins, which in accordance with previous opinions that core proteins determine the function of protein complexes.

As a whole, the identification of protein complexes is of great importance in understanding the cellular organization and functions. Proteins that fall into the same group tend to function similarly, which allows the function of newly discovered proteins to be predicted by identifying protein complexes in which they belong. Since proteins rarely function alone, but rather combine together with other proteins to form protein complexes that execute specific functions. Thus, the loss of particular proteins from a protein complex may be associated with a disease. In this context, our GECluster algorithm may prove useful by aiding disease diagnosis.

## Conclusions

In this paper, a novel protein complex prediction algorithm named GECluster was proposed to detect protein complexes from PPI networks. GECluster was applied on a combined PPI network which is a weighted PPI network constructed from dynamic PPI networks. Dynamic PPI networks were refined from static PPI networks by using gene expression datasets. GECluster was first applied to a training set to determine the optimal parameter value. With this parameter value, GECluster was applied to seven combined PPI networks constructed using data of yeast response to stress throughout a wine fermentation process and gave more accurate protein complex prediction as compared to two classical methods (MCODE and CFinder). The results indicate that using combined PPI network can efficiently improve the protein complex prediction accuracy, which is due to the fact that, unlike classical methods, we introduced dynamic PPI networks and combined PPI networks to take into consideration the biological role of proteins. Dynamic PPI networks are constructed in such a way as to include only those proteins that are translated at a specific time point. The combined PPI network is constructed based on the dynamic PPI network and GO annotations of proteins. By using GO Slims to annotate each protein in the dynamic PPI networks, proteins with similar functions almost always fall into the same GO term defined group, leading to much higher accuracy. The potential of the GECluster algorithm to be employed in protein function prediction may, furthermore, prove useful in aiding the diagnosis of some protein-related diseases.
